# Implications of inappropriate prescription of oral nutritional supplements on the quality of life of cancer outpatients: a cross-sectional comparative study

**DOI:** 10.1007/s00520-022-06837-6

**Published:** 2022-01-25

**Authors:** Islam A. Hassanin, Reem F. M. Salih, Marwa H. M. Fathy, Eman A. Hassanin, Dina H. Selim

**Affiliations:** 1 Faculty of Pharmacy, Alexandria University, Alexandria, 21521 Egypt; 2 Educational and Medical Complex, Al Mawasah University Hospital, Alexandria University, Alexandria, 21526 Egypt

**Keywords:** Oral nutritional supplements, Cancer, Malnutrition, Quality of life, Screening, Role functioning

## Abstract

**Background and aims:**

Oral nutritional supplements (ONS) are considered a cornerstone in the treatment plan of malnutrition in cancer patients. However, the prevalence of inappropriate prescription of ONS is high. In this study, we aim to investigate the effect of inappropriate oral nutritional supplementation (consisting of prescription of ONS without evident clinical indication, or the absence of ONS when at risk of malnutrition) on the quality of life of cancer outpatients.

**Methods:**

A cross-sectional comparative study was conducted in 104 cancer outpatients, receiving ONS without prior malnutrition risk screening (*n* = 51), and patients not receiving ONS (*n* = 53). Nutritional risk screening was performed using the abridged patient-generated subjective global assessment (ab-PG-SGA). The quality of life was assessed using EORTC QLQ-C30 version 3.0 questionnaire. Multivariate analysis was conducted to determine the predictors of quality-of-life scales. Age (years), malnutrition (ab-PG-SGA scores), BMI (kg/m^2^), TSF (mm), MUAC (cm), ONS (yes, no) were entered into the linear regression analysis as predictors (backward stepwise linear regression analysis).

**Results:**

The prevalence of malnutrition risk (ab-PG-SGA ≥ 6) was 74%. The median score of the ab-PG-SGA for ONS receiving group was significantly higher (*p* = 0.045). Furthermore, univariate analysis showed that the scores of the global health status (QoL) and the role functioning (RF) scales were significantly lower for the ONS receiving group (*p* = 0.020 and *p* = 0.016, respectively). Multivariately, malnutrition, inappropriate ONS prescription, and triceps skin fold were found to be predictors of the RF scale, while malnutrition was the only predictor for the QoL.

**Conclusion:**

The inappropriate ONS prescription does not improve nutritional status or quality of life of cancer outpatients.

## Introduction

Malnutrition is considered a prognostic factor, which affects cancer patients [1]. The prevalence of malnutrition in cancer patients varies considerably from 15 to 80% [2]. Furthermore, the rate of mortality based on tumor-related malnutrition ranges between 20 and 30% [3]. Malnutrition possesses a negative impact on the quality of life of cancer patients [4, 5]. It is demonstrated that malnutrition is an independent predictor of poor quality of life in cancer patients on several levels: physical, functional, emotional, and social [5, 6]. For instance, malnutrition was correlated with poor physical functioning in oral or oropharyngeal cancer patients[7]. Thus, the European Society for Clinical Nutrition and Metabolism (ESPEN) recommends malnutrition screening, as well as nutritional intervention for malnourished cancer patients or patients at risk of malnutrition [3]. Nutritional screening can be implemented using non-invasive tools, e.g., the abridged patient-generated subjective global assessment (ab-PG-SGA). This tool is derived from the PG-SGA developed by Ottery (1996) [8]. It was found that the ab-PG-SGA is a valid tool for detection of malnutrition risk in cancer outpatients at a score ≥ 6 [9].

The intervention procedures recommended by ESPEN guidelines consist of nutritional counseling as well as adding oral nutritional supplementation, to increase the oral intake by cancer patients [3]. Oral nutritional supplements (ONS) are homogenous mixtures of balanced nutrients for enhanced caloric intake, commercially available for oral administration [10, 11]. The general criteria for ONS prescription encompass (1) nutritional risk screening or nutritional assessment; (2) identification of the possible etiology of malnutrition; (3) describing the goals of ONS prescription and establishing the desired outcome. These processes should be followed by monitoring and further assessment of the ONS current use, combined with dietary advice [12, 13]. ONS were found to effectively increase the oral intake and to enhance the quality of life in cancer patients [14–17]. However, the prevalence of inappropriate prescription of ONS seems to be high (30–70%) [18–21]. On the other hand, the inappropriate prescription of ONS may fall into three categories: over-prescription, mis-prescription, and under-prescription [12, 22]. Over-prescription involves prescription of ONS without evident clinical indication, while mis-prescription includes overdoses or clinically irrelevant extended duration of treatment of ONS. Finally, under-prescription is manifested as the absence of ONS therapy with evident clinical indication[22]. Furthermore, assessment of ONS intake is significant and challenging. One study concluded that the compliance of cancer patients was low for ONS intake, and therefore, it may affect patients’ weight loss. Hence, it was suggested that education strategies should be implemented for patients receiving ONS [23].

The relationships between inappropriate ONS prescription, the prevalence of malnutrition, and quality of life in cancer outpatients are under-studied. To our knowledge, our study is the first, aiming to investigate the relationship between the inappropriate prescription of ONS, nutritional status, and the quality of life of cancer outpatients.

## Patients and methods

### Patients

A single-centered cross-sectional comparative study was conducted to compare the effect of inappropriate ONS prescription pattern in two groups (receiving or not receiving ONS). Two groups were included in this study:

Group 1: Patients inappropriately prescribed ONS (without malnutrition risk screening prior to inclusion in the study).

Group 2: Patients who did not receive any nutritional support.

All cancer outpatients (≥ 18 years, able to communicate, answer the study questions, and tolerate measurements) at Ayade El-Mostaqbal Hospital were approached to participate in this study in the period between February and April 2021. Patients who were on enteral or parenteral nutritional support were excluded.

### Outcome measures

#### Demographics and patterns of ONS prescription

Demographic data of the patients including age and gender were collected by the researcher through patients’ interviews. Other medical data, e.g., type of cancer, and biochemical tests’ results were collected from the hospital records, retrospectively.

To identify the patterns of ONS prescription, a face-to-face interview with the patients was conducted to assess the nutritional status and a “Yes/No” response on whether they were prescribed any nutritional supplement (NS). When applicable, we identified a pattern of prescription of ONS: non-selective to nutritional status (disease-related malnutrition, i.e., no nutritional assessment) and without a dietitian referral, prior to inclusion to the study. Rather, other medical reasons were reported, e.g., loss of appetite, frailty, aging, and the preference of the patient. In our study, this prescription pattern was referred to as “Inappropriate ONS.” The ONS prescribed by the physician prior to this study was either a powdered form or a drink form, with variable contents. The prescription was based on either forms or both in conjunction.

#### Nutritional screening and anthropometric measurements

Nutritional screening and anthropometric measurements were conducted following the initiation of the study. To identify patients at risk of malnutrition, the abridged patient-generated subjective global assessment (ab-PG-SGA) was utilized. The ab-PG-SGA is considered an applicable screening tool in the oncological outpatient setting. The ab-PG-SGA is a derivative of the PG-SGA tool, including the initial four questions of the PG-SGA with a total score ranging from 0 to 35. Higher scores indicate risk of malnutrition. An ab-PG-SGA score of ≥ 6 was considered to be at risk of malnutrition [9]. In addition, anthropometric measurements, including mid-upper arm circumference (MUAC) measured using a measuring tape, and triceps skin fold (TSF) measured using a TSF caliber on the posterior surface of the arm, were collected. Body mass index (BMI) was calculated as the weight in kilograms divided by the square of height in meters.

#### Quality of life

EORTC QLQ-C30 version 3.0 questionnaire developed by the European Organization for Research and Treatment of Cancer (EORTC) was used to assess the quality of life and compare the group non-selectively receiving ONS and the patients who did not receive any NS. The EORTC QLQ-C30 version 3.0 questionnaire is composed of 30 items: five functional scales, the global quality-of-life (QoL) scale, three symptom scales, and six single items of symptoms commonly reported by oncological patients. The scores were linearly transformed ranging from 0 to 100. Higher scores on the global QoL and the functional scales represent a higher quality of life and higher functioning level, respectively. On the other hand, lower scores on the symptom scale or symptom items represent a lower level of the reported symptoms [24].

### Sample size

A convenient sample size (104 cancer outpatients) was obtained based on the prevalence of the administration of oral nutritional supplements (ONS) in cancer outpatients. Based on a literature search of previous studies, the prevalence of oncological patients receiving ONS was 31.8%, while oncological patients not receiving any nutritional support (NS) was estimated to be 60.2% [25]. The sample size estimation for this cross-sectional comparative study design was based on an effect size (Cohen’s *h*) of 0.578, 95% confidence level (two sided, *α*/*P* = 0.05, *β* level = 0.80, and considering 10% of the patients to drop-out of the study), yielding a requirement of minimum 49 patients in each group [26, 27].

### Statistical analysis

The analysis of the collected data was conducted using Statistical Package for Social Sciences (SPSS, Inc., Chicago, IL, USA) version 23.0. Data are presented as percentages and mean ± standard deviation. Independent samples *t* test was used to compare the continuous variables between the two groups (ONS and non-ONS receiving patients). Alternatively, the Mann–Whitney *U* test was implemented to compare between groups in the case of non-normally distributed data. Pearson’s chi-squared test was used to compare groups for categorical variables. Scores of the EORTC QLQ-C30 scales and items were compared between ONS and non-ONS receiving patients using the Mann–Whitney *U* test.

Linear regression analysis was conducted to investigate the correlation between malnutrition risk and the components (scales and items) of the EORTC QLQ-C30. Multivariate regression analysis was conducted to investigate the potential predictors of the EORTC QLQ-C30 scales, which relates significantly to the inappropriate use of ONS. Multivariately, age (years), malnutrition (ab-PG-SGA scores), BMI (kg/m^2^), TSF (mm), MUAC (cm), ONS (yes, no) were entered into the linear regression analysis as predictors (backward stepwise linear regression analysis), where entry and removal criteria were *p* < 0.05 and *p* > 0.10, respectively. A statistically significant result was set at *P*-value ≤ 0.05 level (two-tailed).

## Results

### Patients’ characteristics

Table 1 shows the baseline characteristics and demographics of the included oncology outpatients. In our study, based on the ab-PG-SGA (score ≥ 6), the prevalence of oncological patients identified at risk of malnutrition was 74%. The patients were 52.7 ± 12.3 years, with the majority of the patients being females (67.3%). Most of the cohort were breast cancer patients (33.7%). The patients were stratified according to the history of ONS use. Patients receiving ONS showed more risk to malnutrition than patients who do not receive any ONS, with a higher median score for ONS receiving group (*p* = 0.045; Table 1).

**Table 1:** Baseline characteristics of 104 cancer patients

Variable	Non-ONS	ONS	*P* value
*N* (%)	53 (51%)	51 (49%)	
Gender, *n* (%)			0.836
Males	18 (33.9%)	16 (31.4%)	
Females	35 (66.0%)	35 (68.6%)	
Age (mean ± SD), years	50.9 ± 11.07	54.5 ± 13.4	0.092
Type of cancer, *n* (%)			
Bladder	0 (0%)	2 (3.9%)	
Bone	1 (1.9%)	4 (7.8%)	
Breast	22 (41.5%)	13 (25.5%)	
Cervical	1 (1.9%)	0 (0%)	
Endometrial	0 (0%)	3 (5.9%)	
Gastrointestinal	14 (26.4%)	13 (25.5%)	
Glioma	0 (0%)	1 (2%)	
Lung	7 (13.2%)	3 (5.9%)	
Lymphoma	3 (5.7%)	3 (5.9%)	
Ovarian	3 (5.7%)	4 (7.8%)	
Prostate	0 (0%)	3 (5.9%)	
Uterine	2 (3.8%)	2 (3.9%)	
Body mass index (kg/m^2^)	29.95 ± 6.52	28.6 ± 6.1	0.294
TSF (mm)	26.2 ± 4.92	24.9 ± 4.1	0.226
MUAC (cm)	34.9 ± 4.5	33.2 ± 4.1	0.085
Screening tool scores, median (range)			
ab-PG-SGA	8 (0.0–17.0)	10 (0.0–22.0)	0.045*
Laboratory markers (mean ± SD)			
ALT (IU/L)	29.9 ± 31.2	20.5 ± 18.7	0.016*
AST (IU/L)	28.4 ± 17.2	20.9 ± 17.7	0.015*
ANC (cells/mm^3^)	6342.2 ± 9402.6	3728.5 ± 3394.2	0.098
Bilirubin (mg/dL)	0.76 ± 0.81	0.55 ± 0.85	0.018*
Creatinine (mg/dL)	0.90 ± 1.25	1.06 ± 1.89	0.376
Platelet count (× 10^3^/µL)	253.9 ± 102.5	278.7 ± 385.6	0.312

*TSF*, triceps skin fold; *MUAC*, mid-upper arm circumference; *ab-PG-SGA*, abridged patient-generated subjective global assessment; *ALT*, alanine aminotransferase; *AST*, aspartate aminotransferase; *ANC*, absolute neutrophil count; *ONS*, oral nutrition supplementation receiving patients; *non-ONS*, patients receiving no ONS

^*^Statistically significant result

### Quality of life

Univariate analysis of the quality of life indicated that the score of QoL of the ONS receiving group (43.9 ± 16.8) was significantly (*p* = 0.020) lower than of the non-ONS receiving group (50.6 ± 14.6). Additionally, the role functioning scale showed a similar trend with a lower score for ONS receiving group (*p* = 0.016; Table 2). On the other hand, there were no significant differences between the two groups in the other components of the EORTC QLQ-C30. Notably, malnutrition risk (ab-PG-SGA score ≥ 6) was significantly correlated with all the components of the quality of life in our cohort, except for the symptom items (diarrhea and constipation) (presented in Table 2).

**Table 2:** The EORTC QLQ-C30 quality of life in oncological patients inappropriately receiving ONS or not receiving ONS and its correlation with malnutrition risk

EORTC QLQ-C30 scale/item	Non-ONS	ONS		Malnutrition risk	
	Mean ± SD (*n* = 53*)*	Mean ± SD (*n* = 51)	*P* value^†^	Standardized *β*	*P* value
***Global health status (QOL)***					
Global health status (QOL)	50.6 ± 14.6	43.9 ± 16.8	0.020*	− 0.268	0.006*
***Functional scale***					
Physical functioning (PF)	48.5 ± 21.9	49.1 ± 21.4	0.753	− 0.441	< 0.005*
Role functioning (RF)	57.9 ± 28.0	44.4 ± 27.6	0.016*	− 0.397	< 0.005*
Emotional functioning (EF)	44.9 ± 28.8	51.5 ± 28.4	0.198	− 0.367	< 0.005*
Cognitive functioning (CF)	58.8 ± 29.3	57.2 ± 31.3	0.817	− 0.441	< 0.005*
Social functioning (SF)	72.6 ± 30.2	63.4 ± 30.2	0.119	− 0.367	< 0.005*
***Symptoms scale***					
Fatigue (FA)	49.5 ± 25.7	53.2 ± 25.4	0.501	0.484	< 0.005*
Nausea and vomiting (NV)	30.2 ± 28.3	28.1 ± 26.8	0.760	0.463	< 0.005*
Pain (PA)	50.3 ± 28.6	53.3 ± 24.8	0.698	0.414	< 0.005*
Dyspnea (DY)	59.7 ± 34.8	54.2 ± 29.8	0.304	0.386	< 0.005*
Insomnia (SL)	58.3 ± 37.8	47.1 ± 30.7	0.086	0.288	0.003*
Appetite loss (AP)	41.5 ± 33.3	46.7 ± 28.6	0.421	0.598	< 0.005*
Constipation (CO)	27.0 ± 31.4	37.3 ± 33.1	0.106	0.123	0.213
Diarrhea (DI)	21.4 ± 30.7	23.5 ± 33.5	0.819	0.178	0.070
Financial difficulties (FI)	30.2 ± 32.9	41.2 ± 29.5	0.059	0.326	0.001*

^*^Statistically significant result

^†^Analyzed by the Mann–Whitney *U* test

Multivariate analysis was conducted to determine the predictors of QoL and the role functioning scale (RF) scores. Malnutrition (*p* < 0.0005), the inappropriate use of ONS (*p* = 0.037), and triceps skin fold (*p* = 0.002) were significantly related to role functioning. Non-significant predictors were age, BMI, and MUAC. However, only malnutrition was significantly related to QoL (*p* = 0.006; Table 3). Age, BMI, TSF, MUAC, and inappropriate ONS use were non-significant as predictors of QoL.

**Table 3:** Stepwise backward multivariate linear regression analysis showing the predictors of the EORTC QLQ-C30 scales in oncological outpatients

EORTC QLQ-C30 scale/predictor	Unstandardized ***B***	SE *B*	95% CI	*P* value
Role functioning scale (RF)				
*Malnutrition*	− 2.413	0.537	− 3.479 to − 1.348	< 0.0005*
*Inappropriate use of ONS*	− 10.716	5.062	− 20.761 to − 0.671	0.037*
*Triceps skin fold*	− 1.728	0.556	− 2.831 to − 0.626	0.002*
*Constant*	122.849	16.089	90.926–154.772	< 0.0005*
Global health status (QoL)				
*Malnutrition*	− 0.902	0.323	− 1.543 to − 0.261	0.006*
*Constant*	55.703	3.310	49.137–62.269	< 0.0005*

^*^Statistically significant result

## Discussion

The results of this study indicated that the prevalence of malnutrition risk in cancer outpatients, based on the ab-PG-SGA, was 74%, which is in line with previously published reports. Silva et al. reported that malnutrition was identified in 71% of cancer patients using PG-SGA [28]. The high prevalence of tumor-related malnutrition reflects poor nutritional status following inappropriate prescription of ONS. Although the mean score of the ab-PG-SGA for patients receiving ONS in our cohort was significantly higher, patients not receiving ONS were presented with an ab-PG-SGA median score > 6, indicating malnutrition risk (Table 1). These results reflect the inappropriate pattern of ONS prescription, which is presented with a combination with under-prescription and over-prescription.

Nutritional intervention, consisting of a dietary advice alone or in combination with an ONS, is considered an important milestone in the treatment of disease-related malnutrition. However, ONS prescription is often complicated with heterogenous reasons, which may compromise its effectiveness, e.g., disregarding nutritional status [12]. It was reported that the pattern of inappropriate ONS prescription is as high as 75% [18]. In our study, we identified an over-prescription, as well as an under-prescription process. The most reported causes for ONS prescription were frailty, aging, and loss of appetite. Moreover, in some circumstances, patients asked for an ONS prescription, in order to increase nutritional intake. Our findings were consistent with the study published by P. Dominguez Castro et al. reporting that about one-third of the general practitioners involved in the prescription of ONS for cancer patients were not adherent to the prescription criteria, and that malnutrition was not prioritized in the prescription process (no nutritional risk screening was conducted) [12]. Therefore, our aim was to evaluate the effect of this prescription pattern on the overall quality of life in cancer outpatients. Despite the positive effect reported previously for ONS on the quality of life of cancer patients [16, 29], our results showed contrary effect in the case of inappropriate ONS prescription. The mean of the QoL scale was lower in ONS receiving group compared to non-ONS receiving group (Table 2). The role functioning scale (RF) was also significantly lower for ONS receiving group (*p* = 0.016). Especially, RF scale possesses a prognostic effect on the overall survival in cancer patients [30]. Malnutrition, inappropriate use of ONS, and TSF were predictors of the RF scale. The inappropriate ONS prescription was negatively related to the RF scale (*B* =  − 10.710; *p* = 0.037). In this regard, the European Society for Clinical Nutrition and Metabolism (ESPEN) guidelines recommend that the prescription of ONS is subjected to cancer patients who are diagnosed as malnourished or at risk of malnutrition. However, the guidelines stated that it was still unclear whether ONS prescription without a dietary counseling could provide an improved clinical outcome [3]. A recently published trial found that the inappropriate ONS prescription alone or in combination with dietary advice did not improve the survival in cancer patients [31]. Additionally, Uster et al. concluded that nutritional intervention including nutritional counseling and oral nutritional supplementation successfully improved energy and protein intake with no improvement of nutritional statuses or quality of life, related to advanced cancer stages [32]. Stemming from our results, we can also infer that the prescription of ONS alone without a dietary advice did not provide any positive effect on the quality of life of cancer outpatients. Although the emotional scale showed an improvement in ONS-treated group, it was not statistically significant.

Interestingly, malnutrition risk was significantly correlated with all the functional scales, as well as symptoms items, except for diarrhea and constipation. These results were similar to previous studies, stating that malnutrition risk is an independent predictor of lower QoL [5]. Subsequently, malnutrition risk was the only predictor of the QoL in our study (*B* =  − 0.902; *p* = 0.006). Several reports have pointed out the negative influence of malnutrition on the quality of life [4], response to treatment [33], complications, and costs [34]. Malnutrition was also found to impact the overall survival in cancer patients [35].

The limitation of the study is manifested in the cross-sectional study design. This design did not allow the quantification of clinical outcome measures. Additionally, the results of the study should be interpreted cautiously. On the other hand, the cross-sectional design offered a sufficient resort to address the conception of the study, and the comparison between groups.

In summary, this study provided insights on the high prevalence of malnutrition in cancer outpatients and the negative influence of inappropriate ONS prescription on the quality of life in cancer patients. The inappropriate use of ONS did not provide any improvements on the quality of life in cancer patients. We recommend the implementation of the general criteria for prescription of ONS for cancer patients, which was included by P. Dominguez Castro et al. [12]. The necessity of dietary advice along with ONS prescription, as well as nutritional follow-up, is considered a major issue to be investigated in future research. Also, it is necessary to raise the awareness of the prescribing the physicians about nutritional risk screening and assessment procedures. Subsequently, it will be imperative to refer patients at risk of malnutrition to a “dietitian” for further assessment and setting a treatment plan.

## Conclusion

The inappropriate pattern of ONS prescription exhibited in cancer outpatient setting does not seem to improve nutritional statuses or quality of life of cancer patients, and the prevalence of malnutrition risk remains high.

## Data Availability

Data is available, and it will be provided upon request to the corresponding author.
